# Effects of Intake of Apples, Pears, or Their Products on Cardiometabolic Risk Factors and Clinical Outcomes: A Systematic Review and Meta-Analysis

**DOI:** 10.1093/cdn/nzz109

**Published:** 2019-10-03

**Authors:** Bridget A Gayer, Esther E Avendano, Emily Edelson, Nanguneri Nirmala, Elizabeth J Johnson, Gowri Raman

**Affiliations:** 1 Gerald J. and Dorothy R. Friedman School of Nutrition Science and Policy, Tufts University, Boston, MA; 2 Institute for Clinical Research and Health Policy Studies, Center for Clinical Evidence Synthesis, Tufts Medical Center, Boston, MA; 3 Jean Mayer USDA Human Nutrition Research Center on Aging, Tufts University, Boston, MA

**Keywords:** apples, pears, cardiovascular disease, cerebrovascular disease, BMI, type-2 diabetes mellitus

## Abstract

Apples and pears contain nutrients that have been linked to cardiovascular health. We conducted a systematic review and meta-analysis to summarize related research. Medline, Cochrane Central, and Commonwealth Agricultural Bureau databases were searched for publications on apple or pear intake and cardiovascular disease (CVD)/ cardiometabolic disease (CMD). Studies in adults (healthy or at risk for CVD) that quantified apple or pear intake were included. Random-effects models meta-analysis was used when ≥3 studies reported the same outcome. In total, 22 studies were eligible including 7 randomized controlled trial, 1 nonrandomized trial, and 14 prospective observational studies. In RCTs, apple intake significantly decreased BMI, but made no difference in body weight, serum lipids, blood glucose, or blood pressure. In observational studies, apple or pear intake significantly decreased risk of cerebrovascular disease, cardiovascular death, type 2 diabetes mellitus, and all-cause mortality. No association was reported for cerebral infarction or intracerebral hemorrhage. In conclusion, apple or pear intake significantly decreased BMI and risk for CVD outcomes.

## Introduction

Fruit intake is associated with a decreased risk of cardiovascular disease (CVD) events and risk factors in several epidemiological studies (1–5). The Dietary Guidelines for Americans 2015–2020 report recommends a high intake of fruit, as part of a healthy eating pattern for the prevention of chronic disease (6). Few Americans eat adequate servings of fruit to adhere to this recommendation; however, apples are the most consumed and the fourth least expensive type of fresh fruit in the United States (7–9). A better understanding of the health effects of apple intake as well as intake of fruits with similar nutrient content, such as pears, aside from total fruit, on CVD could be useful in informing federal nutrition guidance regarding fruit, considering apples’ popularity and accessibility.

CVD risk factors and events are highly prevalent in the United States, and the health and financial burdens of this disease, warrant the investigation of prevention through diet (10). Each year, nearly 1 in every 6 US healthcare dollars is spent on treatment for CVD (11). Direct healthcare costs attributable to CVD were $193 billion and the associated costs due to productivity loss were $123.5 billion in 2012 (11). The health benefits associated with fruit consumption could result in considerable cost savings, through possible reductions of medications, invasive interventions, and lost productivity.

Apples and pears contain several bioactive compounds, including flavonoids, dietary fiber, and antioxidants, that have been individually associated with decreased risk for CVD risk factors and events (12–15). Intakes of dietary fiber and antioxidants from fruits have been found to be significantly associated with a decreased risk for CVD (14–17). In an analysis of the Cancer Prevention Study II, men and women with the highest intake of flavonoids had an 18% risk reduction for CVD mortality (12).

While the cardiovascular health benefits of the bioactive compounds found in apples and pears are widely recognized, the effects of intakes of whole apples and pears and their products has remained somewhat inconclusive. Several studies have found significant CVD risk reductions due to apple or pear intake (16, 18–21), but not all studies (22). A recent systematic review and meta-analysis of observational studies indicated a protective relationship between apple and pear intake and risk of type 2 diabetes mellitus (T2DM), a highly prevalent and costly risk factor for CVD (23). To the best of our knowledge, a systematic review and meta-analysis on apple and pear intake and CVD has not been previously conducted, and this study aims to fill this gap in the literature.

## Methods

We conducted a systematic review of published literature evaluating the effects of apple intake on CVD and cardiometabolic disease (CMD) risk factors and events. We developed a single causal pathway, or analytical framework, depicting the potential association between intake of apples, pears, and their products and CVD/CMD risk factors and outcomes to guide our review (**Supplemental Figure 1**). The systematic review results were reported according to the Preferred Reporting Items for Systematic Reviews and Meta-Analysis statement (24).

### Data sources and study eligibility

We conducted a comprehensive literature search in MEDLINE, Cochrane Central, and Commonwealth Agricultural Bureau abstracts from 1946 through August 2019 for publications that measured apple and/or pear intake and CVD/CMD clinical outcomes and risk factors in adults (**Supplemental Table 1**). No language restriction was applied during searches. Citations were screened in duplicate using the predefined study eligibility criteria and discrepancies were resolved by consensus in group conferences.

#### Study inclusion criteria

We included prospective cohorts and intervention trials conducted in adults ≥18 y of age that quantified the amount of apple or pear intake. We included studies that examined pear or combined apple and pear intake because of similarities in nutrient content between apples and pears and because apple and pear intake is often combined in analysis of nutrition data (51). Studies were eligible if the population was either healthy or had CVD/CMD risk factors (i.e., hypertension, hyperlipidemia, metabolic syndrome, or diabetes) at baseline. Studies with no apple or pear intake or low intake of apples or pears as comparators were accepted. The clinical outcomes of interest included any CVD, acute coronary syndrome, myocardial infarction, ischemic heart disease, stroke, coronary artery disease, atrial fibrillation, all-cause mortality, cardiovascular mortality, cerebrovascular disease, cerebral infarction, and heart failure. CMD risk factors of interest included metabolic syndrome, T2DM, blood glucose, hypertension, systolic blood pressure (SBP), diastolic blood pressure (DBP), hypercholesterolemia, blood lipids [total cholesterol, high density lipoprotein (HDL) cholesterol; low density lipoprotein (LDL) cholesterol , very-low density lipoprotein (VLDL) cholesterol , triglycerides, LDL:HDL cholesterol , and total:HDL cholesterol ), body weight, BMI, and waist-to-hip ratio.

#### Study exclusion criteria

We excluded studies that evaluated apple pectin or apple pomace. We also excluded studies in children and pregnant women. The following study designs were excluded: retrospective, cross-sectional, case reports, and single-arm (interventions with no control group), mixed intervention, pharmacokinetic, in vitro, and cell-culture studies. In addition to the above common eligibility criteria, we established additional criteria specific to the study design.

#### Eligibility criteria for intervention trials

In our analysis of intervention trials, we included studies with known doses of apples, pears, and their products. The minimum intervention duration was at least 1 wk for recorded blood glucose levels and at least 3 wk for other risk factor outcomes. Studies with <5 subjects per arm were excluded.

#### Eligibility criteria for cohort studies

In our analysis of cohort studies, we included studies with reported intake of apple, apple products, pear, pear products, or combined apple and pear. Studies that reported multivariable results adjusted for any potential confounders were eligible. Studies with at least 6 mo of follow-up time were included.

### Data extraction and quality assessment

Data from each study were extracted independently by 1 of 6 investigators and reviewed and confirmed by ≥1 other team member. Any conflicts regarding extraction were resolved in team discussions. The extracted data table included study design, intervention description, population characteristics, methods for controlling for potential confounders or effect modifiers, outcomes, and results depicting associations between apple or pear intake and the specific outcome(s) of interest.

We assessed the methodologic quality of each study based on predefined criteria, in accordance with the Agency for Healthcare Research and Quality's suggested methods for systematic reviews (25). Study quality was determined in duplicate and discrepancies were resolved by consensus in group discussion. We applied risk of bias items in the modified Newcastle–Ottawa Scale (26) for observational studies, the Cochrane risk of bias for clinical trials (27), and nutrition-specific items for a critical appraisal of micronutrient systematic reviews for both clinical trials and observational studies (28).

### Data synthesis

Analyses were conducted separately for intervention trials and observational studies. We performed random-effects model meta-analyses when similar data from ≥3 studies were available (29). For intervention trials, we combined net differences [net change = (apple intake_final_ − apple intake_initial_) − (control_final_ − control_initial_)] for continuous outcomes. We tested between-study heterogeneity with the *Q* statistic (significant when *P* < 0.10) and quantified its extent with *I^2^*. *I^2^* values of 25%, 50%, and 75% were considered low, moderate, or high heterogeneity, respectively (30).

For observational studies, we synthesized RRs comparing the extreme categories of apple or pear intake (highest compared with lowest, as defined within each study) provided that the categories corresponded to similar doses of intake across studies. We performed sensitivity analyses when there were studies reporting various similar doses of apple or pear intake (including a comparison of medium intake to low intake) or if they reported data for subgroups, such as sex. All statistical analyses were conducted in Stata version 14 (StataCorp). Forest plots were created using R version 3.3 (R Core Team).

For studies that reported quantities by serving size, we considered a serving equivalent to 1 medium-sized apple or pear. For a study that reported apple intake as catechin content, we used the USDA Food Composition Database (51) to obtain the total catechin composition of a medium-sized apple and back calculated the amount of apple consumed for each quartile.

All included studies that could not be combined quantitatively in meta-analyses were summarized in narrative form and in tables that tabulated the important features of the study populations, design, intervention, outcomes, and results. Summary tables were organized by outcomes of interest.

## Results

**FIGURE 1:**
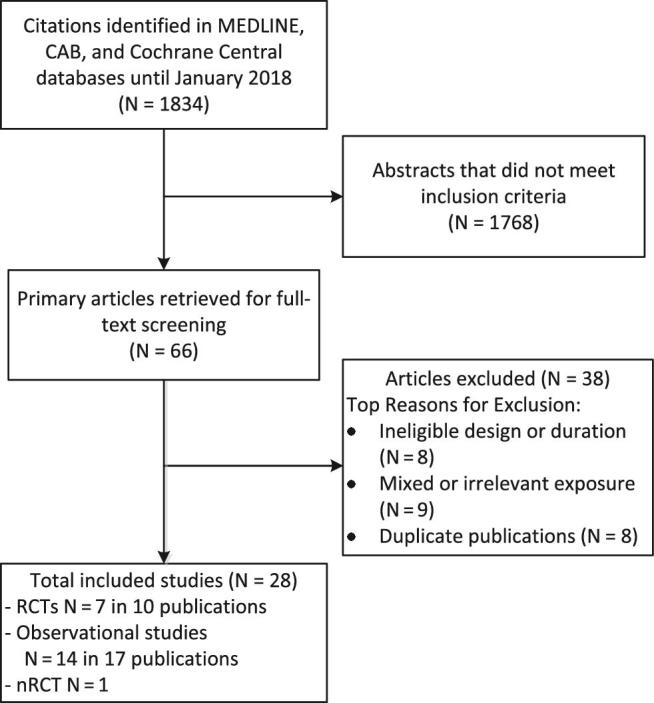
Results of comprehensive literature search.

Database searches identified 1834 citations. Full-text articles of 66 citations that were accepted in abstract screening were retrieved and reviewed against eligibility criteria. Full-text screening yielded 28 eligible articles (**Figure 1**). Of 28 eligible articles, 11 reported intervention trial data, and 17 reported prospective cohort data. No foreign language publications met the inclusion criteria.

### Intervention trials

Eight intervention trials (7 RCTs and 1 nonrandomized trial) in 11 publications that enrolled 596 participants were eligible. Trials were conducted in Brazil (*n* = 1), Denmark (*n* = 1), Germany (*n* = 1), Iran (*n* = 1), Ireland (*n* = 1), Norway (*n* = 1), and the United States (*n* = 1), and for 1 trial the location was not reported. The duration of the trials ranged from 4 to 20 wk. Two trials included only men, 3 included only women, and 3 trials included both men and women. Of 7 RCTs, 5 were parallel-arm randomized trials, and 2 were crossover trials. Included participants in RCTs varied considerably; they were healthy (31, 32), nondiabetic (33), or had increased risk of CVD/CMD (19, 32, 34, 35). The apple or pear interventions and their comparators varied across RCTs. Apple or pear intake ranged from 75 to 900 g/d, and comparators included no apple or pear, control beverages with equivalent amounts of calories and fructose, dried plums, oat cookies, and kiwis. In addition to 12-wk time points, long-term data at 6 and 12 mo were reported for 1 RCT. Meta-analyses of RCTs were conducted for the following outcomes: body weight [5 RCTs (31–34, 36)], BMI [3 RCTs (31–33)], HDL cholesterol [4 RCTs (19, 31, 33, 36)], total cholesterol (TC) [5 RCTs (19, 32–35)], LDL cholesterol [4 RCTs (19, 31, 33, 36)], and triglycerides (TG) [5 RCTs (19, 31, 33, 36)]. No meta-analysis was conducted for SBP, DBP, waist circumference, glucose, or waist:hip, LDL:HDL cholesterol, total cholesterol:HDL cholesterol, or glucose:insulin ratios, as these outcomes were reported in <3 RCTs. Baseline details for intervention trials can be found in **Table 1**, meta-analysis results are tabulated in **Table 2**, the risk of bias of each study is listed in **Supplemental Table 2**, and meta-analysis results of trials reporting the effect of apple in percentage net change are tabulated in **Supplemental Table 3**.

**TABLE 1:** Baseline study and participant characteristics of intervention studies^1^

Author and y	Country (funding source)	Study design (duration)	Subjects enrolled (analyzed), *n*	Male, %	Mean age, y	BMI, kg/m^2^	Comorbidities, %	Apple/pear, daily dose	Diet comparison, daily dose	Outcomes
Barth 2012	Germany (NR)	RCT-P (4 wk)	68 (68)	100	49	30.84	Healthy: NRDiabetes: 0Hyperlipidemia/dyslipidemia: NRHypertension: NRExisting CVD: NR	Apple juice, 2250 mL	Controlled beverage, 2250 mL	BMI, body weight, HDL, LDL, TC, TG, waist circumference
Chai 2012; Hooshmand 2013^2^	US (G)	RCT-P (1 y)	160 (90)	0	56.65	24.5	Healthy: 100Diabetes: 0Hyperlipidemia/dyslipidemia: 0Hypertension: 0Existing CVD: 0	Dried apple, 75 g	Dried plums, 100 g	BMI, body weight, HDL, LDL, LDL:HDL, TC, TC:HDL, TG
De Oliveira 2003, 2008	Brazil (G, A, I)	RCT-P (12 wk)	51 (35)	0	44.03	31.87	Healthy: 0Diabetes: 0Hyperlipidemia/dyslipidemia: 100Hypertension: NRExisting CVD: NR	Apple or pear, 900 g	Oatmeal cookie, 180 g	Blood glucose, BMI, body weight, glucose, glucose:insulin, insulin, TC, TG
Gormley 1977	Ireland (I)	nRCT-P (16 wk)	80 (76)	100	NR	NR	Healthy: NRDiabetes: NRHyperlipidemia/dyslipidemia: NRHypertension: NRExisting CVD: NR	2 additional apples per d to their diet	≤3 apples or apple-like fruits/wk	TC, HDL
Navaei 2017; Johnson 2016	NR (Fresh Pear Committee; Pear Bureau Northwest)	RCT-C (12 wk)	50 (43; 36)	NR	NR	NR	Healthy: 0Diabetes: NRHyperlipidemia/dyslipidemia: NRHypertension: NRExisting CVD: NR	Pear, 178 g/d	50 g pear-flavored drink mix placebo	SBP, DBP, waist:hip
Ravn-Haren 2013	Denmark (G)	RCT-C (20 wk)	23 (23)	39	36.2	22.3	Healthy: 100Diabetes: 0Hyperlipidemia/dyslipidemia: 0Hypertension: 0Existing CVD: 0	Whole apple, 500 g; cloudy apple juice, 500 mL; clear apple juice, 500 mL; apple pomace, 22 mg	Only restricted diet allowed	Body weight, DBP, HDL, Insulin, LDL, SBP, TC, TC:HDL, TG, waist:hip
Svendsen 2015	Norway (F)	RCT-P (8 wk)	118 (115)	42.37	55	26	Healthy: 0Diabetes: NRHyperlipidemia/dyslipidemia: NRHypertension: 100Existing CVD: 0	Apple, 170 g/d	Green kiwi fruit, 360 g	Body weight, DBP, SBP
Vafa 2011	Iran (A)	RCT-P (8 wk)	46 (46)	100	41.37	26.87	Healthy 0Diabetes: 0Hyperlipidemia/dyslipidemia: 100Hypertension: NRExisting CVD: 0	Apple, 300 g/d	No apple, 0 g	ApoB, HDL, LDL, LDL:HDL, Lp(a), TC, TG, VLDL

1 A, academic; ApoB, apolipoprotein B; CVD, cardiovascular disease; DBP, diastolic blood pressure; F, foundation; G, government; HDL, high density lipoprotein; I, industry; LDL, low density lipoprotein; Lp(a), lipoprotein(a); NR, not reported; RCT-C, randomized control trial, cross-over; RCT-P, randomized control trial, parallel; SBP, systolic blood pressure; TC, total cholesterol; TG, triglycerides; VLDL, very low density lipoprotein.

2 Chai 2012 and Hooshmand 2013 and De Oliveira 2003 and De Oliveira 2008 were included as one row each in the above table since they drew data from the same trial.

**TABLE 2:** Meta-analysis of intervention trials reporting the effect of apple compared with control on serum lipids and body composition^1^

		Net change (95% CI) (*I*^2^)		
	Studies (subjects), *n*	Analysis 1	Analysis 2	Analysis 3
TC (mg/dL)	5 (324)	−4.10 (−13.02, 4.82) (46.0%)	−5.14 (−16.31, 6.03) (64.7%)	−4.50 (−14.45, 5.45) (54.8%)
LDL (mg/dL)	4 (289)	−4.75 (−10.40, 0.90) (19.0%)	−7.09 (−15.54, 1.35) (61.4%)	−5.29 (−11.77, 1.18) (34.4%)
HDL (mg/dL)	4 (289)	−0.79 (−2.58, 0.99) (0.0%)	−0.97 (−2.76, 0.82) (0.0%)	−0.75 (−2.55, 1.05) (0.0%)
TG (mg/dL)	5 (324)	8.91 (−10.32, 28.14) (59.1%)	9.66 (−9.05, 28.36) (54.0%)	8.60 (−14.93, 32.13) (72.4%)
Body Weight (kg)	5 (393)	0.14 (−0.45, 0.73) (0.0%)	0.14 (−0.45, 0.73) (0.0%)	0.14 (−0.45, 0.73) (0.0%)
BMI (kg/m^2^)	3 (229)			**−0.39 (−0.59, −0.20) (0.0%)**

Meta-analyses were conducted using the random-effects model. Analysis 1: for one 3-arm parallel trial Chai 2012, the main analyses included 3-mo intake time-point because it was the closest to the final time points in the other studies. Analysis 2: for one 3-arm parallel trial Chai 2012, sensitivity analysis was conducted using the 6-mo time point. Analysis 3: For one 3-arm parallel trial Chai 2012, sensitivity analysis was conducted using the 12-mo time point. *I*^2^ is an indicator of between-comparison heterogeneity. *I*^2 ^>50% was deemed as having significant heterogeneity. HDL, high-density lipoprotein cholesterol; LDL, low-density lipoprotein cholesterol; TC, total cholesterol; TG, triglycerides.

### Body weight–related outcomes

#### Body mass index

**FIGURE 2:**
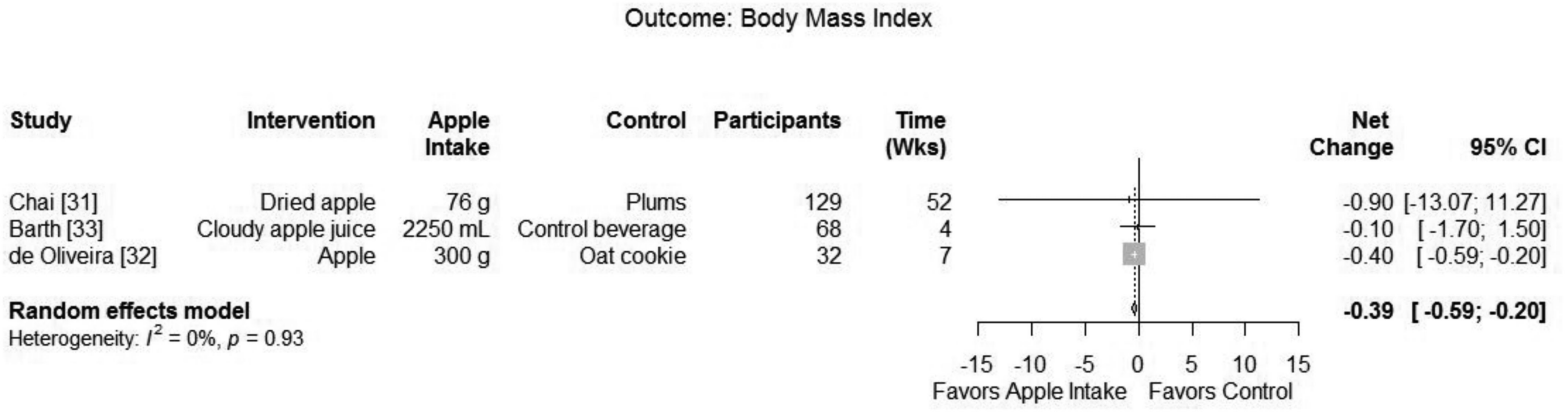
Meta-analysis of intervention trials reporting the effect of apple consumption compared with control on BMI (kg/m^2^).

Three RCTs with a total of 229 participants (31–33) reported the effect of apple intake on BMI. Meta-analysis of the 3 trials comparing apple with a variety of controls found a significant decrease in BMI (summary net change:−0.39; 95% CI: −0.59, −0.20) without heterogeneity (*I*^2^ 0.0%; *P* = 0.934) (**Figure 2**).

#### Body weight

Five trials including a total of 393 participants (31–34, 36) reported the effect of apple intake on body weight. Meta-analysis of the 5 trials, which compared apple with different controls, found no difference in body weight (summary net change: 0.14; 95% CI: −0.45, 0.73; *I*^2^ = 0.0%; *P* = 1.00) between groups. Sensitivity analysis using long-term (6-mo and 12-mo results) data found similar results (31).

#### Waist-to-hip ratio

One crossover RCT conducted in Denmark in 23 healthy men and women (36) measured the effect of apple intake on waist-to-hip ratio and found no difference between groups. A separate crossover RCT conducted among 43 adults at risk for CVD reported the effect of pear intake on waist-to-hip ratio (35). Waist-to-hip ratio in the group consuming pears was significantly reduced compared to that of the control group at both 6- and 12-wk time points.

#### Serum lipid outcomes

##### Total cholesterol

**FIGURE 3:**
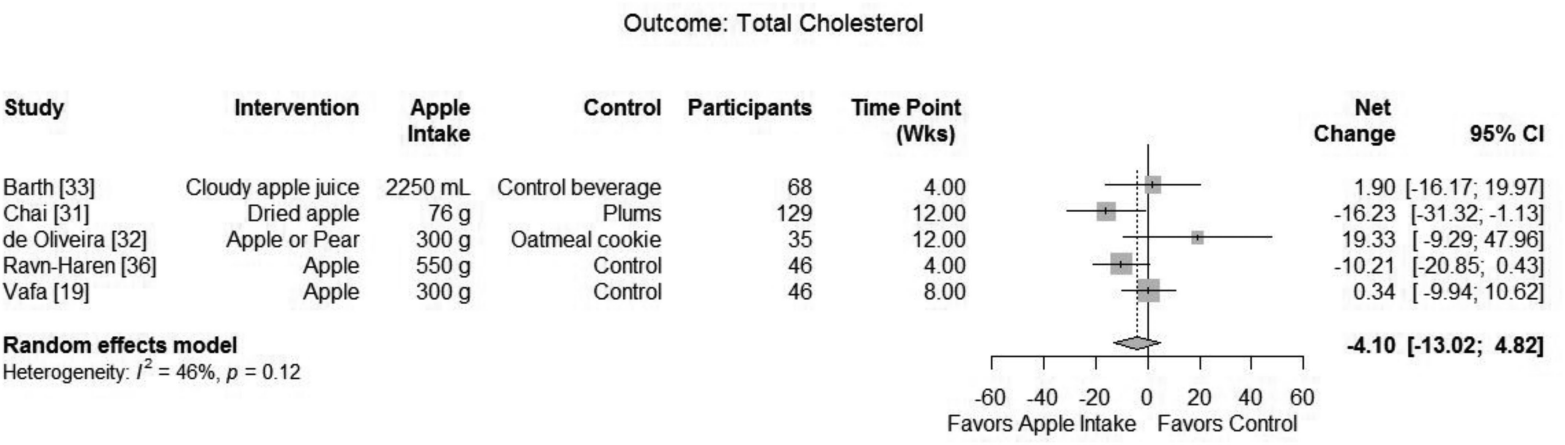
Meta-analysis of intervention trials reporting the effect of apple consumption compared with control on total cholesterol.

Five RCTs (19, 31–33, 36) and 1 nonrandomized trial (37) investigated the effect of apple intervention on serum total cholesterol. Meta-analysis of 5 RCTs with a total of 324 subjects found no effect on total cholesterol (summary net change: −4.10; 95% CI: −13.02, 4.82) with a moderate heterogeneity (*I*^2^ = 46.0%, *P* = 0.116) (**Figure 3**). Sensitivity analysis using the additional time points of 6 mo (net change: −5.14; 95% CI: −16.31, 6.03; *I*^2 ^= 64.7%) and 12 mo (net change: −4.50; 95% CI: −14.45, 5.45; *I*^2 ^= 54.8%) also found no effect of apple on serum total cholesterol.

In the single nonrandomized trial of 70 men that used a time point of 16 wk, there was a significantly lower total cholesterol in the apple intake group versus the control group (37).

##### HDL cholesterol

Four RCTs (19, 31, 33, 36) and 1 nonrandomized trial (37) reported on the effect of apple intake on serum HDL cholesterol concentrations. Meta-analysis of the 4 RCTs with a total of 289 participants found no difference in HDL cholesterol between apple intake and control groups (net change: −0.79; 95% CI: −2.58, 0.99; *I*^2 ^= 0.0%; *P* = 0.726). Sensitivity analyses using 6-mo (net change: −0.97; 95% CI: −2.76, 0.82; *I*^2 ^= 0.0%; *P* = 0.817) and 12-mo time points (net change: −0.75; 95% CI: −2.55, 1.05; *I*^2 ^= 0.0%; *P* = 0.647) also found no difference in HDL cholesterol between apple intake and control groups (31).

In the single nonrandomized trial of 70 men that compared consumption of 2 apples per d with consumption of 3 apples per wk for 16 wk found a significant increase in HDL cholesterol with apple intake compared with control (38).

##### LDL cholesterol

Four trials (19, 31, 33, 36) that included a total of 175 participants reported on the effect of apple intake on LDL cholesterol. The main meta-analysis found no difference in LDL cholesterol (summary net change: −4.75 mg/dL; 95% CI: −10.40, 0.90; *I*^2 ^= 19.0%) between apple intake and control. In sensitivity meta-analyses using 6-mo (summary net change: −7.09; 95% CI: −15.54, 1.35; *I*^2 ^= 61.4) and 12-mo time points (summary net change: −5.29; 95% CI: −11.77, 1.18; *I*^2 ^= 34.4%) found no difference in LDL cholesterol (31).

##### Triglycerides

Five RCTs (19, 31–33, 36) reported the association between apple intake and TG. A meta-analysis of RCTs with a total of 324 subjects compared apple intake with a heterogeneous control group and found no difference in TG (net change: 8.91 mg/dL; 95% CI: −10.32, 28.14; *I*^2 ^= 59.1%, *P* = 0.044). Sensitivity analysis using 6-mo (net change: 9.66 mg/dL; 95% CI: −9.05, 28.36; *I*^2^ = 54.0%) and 12-mo time points (net change 8.60 mg/dL; 95% CI: −14.93, 32.13; *I*^2 ^= 72.4) found no difference in LDL cholesterol between groups (31).

##### Other serum lipid outcomes

One parallel RCT (19) reported the effect of apple intake for 8 wk on VLDL cholesterol in 46 hyperlipidemic, overweight men. This RCT found that there was no difference in VLDL cholesterol between apple and control groups. Two RCTs (19, 31) that reported on the effect of apple intake on LDL cholesterol: HDL cholesterol found no significant difference between groups.

##### Total cholesterol: HDL cholesterol

Two RCTs (31, 36) that included a total of 206 healthy participants found no difference in total cholesterol: HDL cholesterol between apple intake compared with control groups.

##### Apolipoprotein B

One RCT (19) that included 46 hyperlipidemic men measured the effect of apple intake on apolipoprotein B. There was no significant difference in apolipoprotein B concentrations between the apple and control groups.

##### Systolic and diastolic blood pressure

Two RCTs (34, 36) reported the effect of apple intake on SBP and DBP. Among 115 hypertensive participants, one RCT (34) found no difference in SBP (net change: −0.6; 95% CI −4.7, 3.5; *P* = 0.825) or DBP (net change: 1.7; 95% CI −0.8, 4.2; *P* = 0.177) between groups.

Another RCT (36), which included 23 healthy men and women, found a statistically significant reduction for both SBP (net change: −3.93 mm Hg; 95% CI: −7.60, −0.26; *P* = 0.04) and DBP (net change: −2.96 mm Hg; 95% CI: −5.97, −0.05; *P* = 0.05) after 4 wk of apple intervention compared with control.

One crossover RCT (35) reported the effect of pear intake on SBP. Among 43 adults at risk for CVD, SBP tended to be reduced at 12 wk in the pear intake group while no changes were observed in the control group. A second publication reporting results for the same RCT (38) reported the effect of pear intake on SBP and DBP. Among 36 adults at risk for CVD, SBP was significantly lower than baseline levels among the pear group, while no changes were observed for the control group. No changes were observed in DBP for either group.

##### Glucose metabolism outcomes

Three RCTs (32, 36, 39) with a total of 236 participants reported the effect of apple intake on insulin levels and found no difference in insulin levels between groups. A meta-analysis was not possible because 1 trial only reported qualitative results (39). Two RCTs (32, 39) that reported the effect of apple intake on blood glucose levels and 1 RCT (32) that reported on glucose:insulin found no difference between groups.

##### Cohort studies

A total of 14 cohorts were evaluated in 17 publications and the following outcomes were examined: acute coronary syndrome [1 cohort (20)], cerebrovascular disease [4 cohorts (17, 21, 40, 41)], diabetes [5 cohorts in 4 studies (42–45)], CVD mortality [3 cohorts (14, 18, 46, 47)], all-cause mortality [3 cohorts (18, 46, 48)], hypertension [3 cohorts in 1 study (49)], and body weight [3 cohorts in 1 study (50)]. Studies were conducted in the United States (*n* = 5), Denmark (*n* = 1), Finland (*n* = 1), Sweden (*n* = 3), The Netherlands (*n* = 2), Australia (*n* = 1), and the United States/China (*n* = 1). Three cohorts reported results for at ≥1 outcome stratified by sex. Six cohorts included only women, 2 included only men, and 7 included both men and women. Three publications reported combined results for Nurses’ Health Study (NHS), Nurses’ Health Study II (NHS II), and Health Professionals Follow-Up Study (HPFS) and stratified their results by cohort. Nine of 14 cohorts reported results for apple and pear intake and the remaining 5 cohorts reported only apple intake. Studies varied in the length of follow-up and in the amount of apple or pear intake (high, medium, or low intake). Follow-up ranged from 4 to 28 y. High apple or pear intake ranged from to 35.2 to more than 100 g/d, medium apple or pear intake ranged from 0 to 100 g/d, and low apple or pear intake ranged from 0 to 35.2 g/d. Baseline details for studies can be found in **Table 3**, the meta-analysis results are tabulated in **Table 4**, and risk of bias is listed in **Supplemental Table 4**.

**TABLE 3:** Baseline study and participant characteristics of cohort studies^1^

Author and y	Country (funding source)	Cohort name	Duration (enrollment y)	Patients enrolled (analyzed), *n*	Male, %	Mean age, y	BMI, kg/m^2^	Comorbidities, %	Apple dose	Outcomes	Model adjustments
Alperet, 2017	US, China (G, A)	Singapore Chinese Health Study	1993–1998 (494,741 person-y = 17 y)	63,257 (45,411)	31	Men: 57; Women: 56	Men: 22.7; Women: 23.2	Healthy: NRDiabetes: 0Hyperlipidemia/dyslipidemia: NRHypertension: 19.2Existing CVD: 0	Never/rarely;<1 serving/wk;1 serving/wk;2–3 serving/wk;4–6 servings/wk;>1 serving/d	T2DM	Diet
Mink 2007; Arts, 2001	US (F,G)	None	1986 (12 y)	34,492 (34,492)	0	61	26.9	Healthy: 89.6–90.7Diabetes: 5.7–6.5Hyperlipidemia/dyslipidemia: NRHypertension: 34.1–34.8Existing CVD: 0	Apples and pears:<1 serving/wk;1 serving/wk;>1 serving/wk	IHD mortality, CVD mortality, Stroke mortality	Age, alcohol intake, cholesterol, diet
Bertoia, 2015	US (G)	NHS I;NHS II;HPFS	NHS: 1976;NHS II: 1989;HPFS: 1986 (4 y)	133,468 (133,468)	14.48	41.65	NR	Healthy: NRDiabetes: 0Hyperlipidemia/dyslipidemia: NRHypertension: NRExisting CVD: 0	Apples and pears:0 servings/d;0.31 servings/d	Body weight	Age, BMI, diet, smoking status, hours of sitting or watching TV, hours of sleep physical activity
Borgi, 2016	US (G, A)	NHS I;NHS II;HPFS	NHS: 1976;NHS II: 1989;HPFS: 1986 (2,939,124 person-y)	187,453 (123,059)	19.6	25–75	NR	Healthy: NRDiabetes: NRHyperlipidemia/dyslipidemia: NRHypertension: 0Existing CVD: NR	Apples and pears:<1 per mo;1–3 per month;1–3 per wk;>4 per wk	Hypertension	Age, alcohol intake, analgesic use, BMI, current oral contraceptive use, ethnicity, hypertension family history, menopausal status, physical activity smoking status, use, weight change
Hansen, 2010	Denmark (G)	Danish Diet, Cancer and Health cohort	1993–1997 (7 y)	53,383 (53,383)	46.95	Median: M 55; F 56	Median: M 26; F 25	Healthy: NRDiabetes: 0Hyperlipidemia/dyslipidemia: NRHypertension: NRExisting CVD: 0	M: < 18 g apple/d;18–98 g apple/d;>98 g apple/dF: <18 g apple/d;18–54 g apple/d;>54 g apple/d	Acute Coronary Syndrome	BMI, diet, education level, smoking, alcohol intake, alcohol abstainers and physical activity, total cholesterol, systolic blood pressure
Hansen, 2017	Denmark (F)	Danish Diet, Cancer and Health cohort	1993–1997 (Median: 13.5 y)	57,053 (55,338)	48	Mean of median: 56.1	Median: 25.5	Healthy: NRDiabetes: NRHyperlipidemia/dyslipidemia: NRHypertension: NRExisting CVD: NR	Apples and pears:M: 0–56 g/d,56 < g/dF: 0–71 g/d,71 < g/d	Total stroke, ischemic stroke, hemorrhagic stroke, and intracerebral hemorrhage	Alcohol intake, BMI, diabetes, diet, education, hypertension, hypercholesterolemia. physical activity, smoking, atrial fibrillation, waist circumference
Hertog, 1993	Netherlands (G)	Zutphen Elderly Men study	1985 (5 y)	805 (805)	100	71.26	25.5	Healthy: NRDiabetes: NRHyperlipidemia/dyslipidemia: NRHypertension: NRExisting CVD: 37.3	0 apples/d;0–1 apples/d;>1 apple/d	Cardiovascular mortality	Age, BMI, diet, and risk factors [includes history of MI in 1985, physical activity, smoking, serum TC and HDL, SBP]
Hodgson, 2016	Australia (G, F, H)	Calcium Intake Fracture Outcome Study	1998 (15 y)	1500 (1456)	0	75.16	27.2	Healthy: NRDiabetes: 6Hyperlipidemia/dyslipidemia:18.52Hypertension: 43.2Existing CVD: 23.27	<5 g/d;5–100 g/d;>100 g/d	CVD mortality	Age, alcohol intake, antihypertensive meds, BMI, cancer, cholesterol lowering meds, CVD, diabetes, diet, low-dose aspirin, physical activity smoking status, socio-economic status, treatment code
Knekt, 1996	Finland (None)	Finnish mobile clinic	1967–1972 (20–23 y)	5133 (5133)	53.5	44.97	25.85	Healthy: NRDiabetes: NRHyperlipidemia/dyslipidemia: NRHypertension: 38.47Existing CVD: 0	M: >54 g; 0 g;F: >71 g; 0 g	Coronary mortality; total mortality	Age, BMI, cholesterol, hypertension, smoking status
Knekt, 2000	Finland (None)	Finnish mobile clinic	1967 (28 y)	9208 (9208)	NR	37.46	NR	Healthy: NRDiabetes: NRHyperlipidemia/dyslipidemia: NRHypertension: NRExisting CVD: 0	M: >54 g; 0 g;F: >71 g; <5 g	Acute stroke; All cerebrovascular disease; Intracerebral hemorrhage; thrombosis embolia	Age, BMI, diet, diabetes, geographical region, hypertension, serum cholesterol, smoking, occupation
Lacoppidan 2015	Denmark (F, G)	Danish Diet, Cancer and Health cohort	1993–1997 (median 15.3 y)	57,053 (55,060)	47.2	Median: M 55; F 56	Median: M 24.8; F 26.1	Healthy: NRDiabetes: 0Hyperlipidemia/dyslipidemia: NRHypertension: NRExisting CVD: NR	Apples and pears:M: 0–56 g/d,56 < g/dF: 0–71 g/d,71 < g/d	T2DM	Age, alcohol intake, BMI, diet, education level, physical activity, smoking, waist circumference,
Larsson, 2013	Sweden (G)	SMC; COSM	1998–2008 (10.2 y)	74,961 (74,961)	53.9	60.3	NR	Healthy: NRDiabetes: 5.96Hyperlipidemia/dyslipidemia: NRHypertension: 20.8Existing CVD: 0	Apples and pears:18 g/d;36 g/d;90 g/d;180 g/d	Cerebral infarction, iIntracerebral hemorrhage, subarachnoid hemorrhage, total stroke	Age, alcohol intake, aspirin use, BMI, diabetes, education, family history of myocardial infarction, history of hypertension, sex, smoking status and pack-years of smoking, physical activity
Oude Griep, 2011	Netherlands (G, I)	MORGEN Study	1993–1997 (10.3 y)	20,069 (20,069)	45	42	24.85	Healthy: 100Diabetes: 0Hyperlipidemia/dyslipidemia: 0Hypertension: 0Existing CVD: 0	Apples and pears:25 g/d increase in intake	Stroke	Age, alcohol intake, BMI, diet, educational level, family history of AMI, hormone replacement therapy use, sex, smoking status
Roswell, 2015	Sweden (G)	Swedish Women's Lifestyle and Health cohort	1991–1992 (21.3 y)	44,961 (44,961)	0	39	23.0	Healthy: NRDiabetes: 0Hyperlipidemia/dyslipidemia: NRHypertension: NRExisting CVD: 0	Apples and pears:<35.2 g/d;≥35.2 g/d	Total mortality	Age, alcohol intake, BMI, current tobacco consumption, diet, education, smoking status, time since smoking cessation
Song, 2005	USA (G)	Women's Health Study	1993 (8.8 y)	38,018 (38,018)	0	53.88	25.8	Healthy: NRDiabetes: 0Hyperlipidemia/dyslipidemia: NRHypertension: 25.08Existing CVD: 0	0/wk;<1/wk;2–6/wk;>1/d	T2DM	Age, alcohol use, BMI, diet, exercise, family history of diabetes history of cholesterol, history of hypertension, smoking
Wedick, 2012	United States (G)	NHS I; NHS II; HPFS	NHS (1984); NHS II (1991); HPFS (1986) (24 y)	NHS: 70,359; NHS II: 89,201; HPFS: 41,334 (3645,585 person-years)	NHS: 0; NHS II: 0; HPFS: 100	NHS: 50; NHS II: 36; HPFS: 53	NR	Healthy: NRDiabetes: 0Hyperlipidemia/dyslipidemia: NRHypertension: 16.77Existing CVD: 0	<1 time/mo; 1–3 times/mo; 1 time/wk; 2–4 times/wk; >5 times/wk	T2DM	Age, BMI, diet, ethnicity, family history of diabetes, hormone use, oral contraceptive use, postmenopausal status, smoking status

1 AMI, acute myocardial iInfarction; IHD, ischemic heart disease; COSM, Cohort of Swedish Men; CVD, cardiovascular disease; F, foundation; G, government; HDL, high density lipoprotein; H, hospital research committee; HPFS, Health Professional Follow-up Study; I, industry; MORGAN, Monitoring Project on Risk Factors and Chronic Diseases in the Netherlands; MI, myocardial infarction; NHS, Nurses’ Health Study; NR, not reported; SBP, systolic blood pressure; SMC, Swedish Mammography Cohort; TC, total cholesterol; T2DM, type 2 diabetes mellitus.

**TABLE 4:** Meta-analysis of observation studies reporting the effect of apple compared with low dose on CVD risk factors and events^1^

	Studies (subjects), *n*	Analysis 1 High dose	Analysis 1 Net change (95% CI)	Analysis 2 High dose	Analysis 2 Net change (95% CI)	Analysis 3 High dose	Analysis 3 Net change (95% CI)	Analysis 4 High dose	Analysis 4 Net change (95% CI)
Cerebrovascular disease/total stroke	3	Knekt 2000, Hansen 2017 (M ≥ 54 g, W ≥ 71 g); Larsson (90 g)	**0.89 (0.83, 0.95)**	Knekt 2000, Hansen 2017 (M ≥ 54 g; W ≥ 71 g); Larsson (180 g)	**0.89 (0.83, 0.95)**				
	(139, 507)		*I* ^2^ = 0.0%		*I* ^2^ = 3.5%				
Cardiovascular death	3	Hertog 1993 (>110 g), Hodgson 2016 (>100 g), Mink (>20 g)	**0.86 (0.78, 0.95)**	Hertog 1993 (19–110 g), Hodgson 2016 (5–100 g), Mink (>20 g)	**0.87 (0.79, 0.96)**				
	(36, 753)		*I* ^2^ = 0.0%		*I* ^2^ = 0.0%				
Thrombosis or embolia or cerebral infarction	2	Knekt 2000 (M ≥ 54 g, W ≥ 71 g); Larsson 2013 (90 g)	**0.76 (0.55, 1.05)**	Knekt 2000 (M ≥ 54 g; W ≥ 71 g); Larsson 2013 (180 g)	0.76 (0.55, 1.06)				
	(84, 169)		*I* ^2^ = 50.4%		*I* ^2^ = 52.3%				
T2DM incidence	4	Alperet 2017, Lacoppidan 2015 (>56, >71), Song 2005 (≥ 180); Wedick (>128.6)	**0.86 (0.77, 0.95)**	Alperet 2017 (51–77 g), Lacoppidan 2015 (>56, >71), Song 2005 (51.4–154.3 g); Wedick (51.4–102.9 g)	**0.88 (0.80, 0.96)**	Alperet 2017, Lacoppidan 2015 (>56, > 71), Song 2005 (≤25.7 g); Wedick (25.7 g)	**0.92 (0.86, 0.99)**	Alperet 2017, Lacoppidan 2015 (>56, > 71), Song 2005 (≤25.7 g); Wedick (6–18 g)	**0.93 (0.87, 0.99)**
	(339, 383)		*I* ^2^ = 78.0%		*I* ^2^ = 84.2%		*I* ^2^ = 74.9%		*I* ^2^ = 69.14%
T2DM incidence (females-only)	3	Alperet 2017, Lacoppidan 2015 (>71), Song 2005 (≥ 80); Wedick (>128.6)	**0.81 (0.68, 0.96)**	Alperet 2017 (51–77 g), Lacoppidan 2015 (>71), Song 2005 (51.4–154.3 g); Wedick (51.4–102.9 g)	**0.86 (0.74, 0.98)**	Alperet 2017, Lacoppidan 2015 (>71), Song 2005 (≤25.7 g); Wedick (25.7 g)	**0.92 (0.82, 1.02)**	Alperet 2017, Lacoppidan 2015 (>71), Song 2005 (≤25.7 g); Wedick (6–18 g)	**0.92 (0.83, 1.02)**
	(293, 867)		*I* ^2^ = 89.7%		*I* ^2^ = 87.9%		*I* ^2^ = 81.34%		*I* ^2^ = 78.7%
Intracerebral hemorrhage	3	Knekt 2000, Hansen 2017 (M ≥ 54 g, W ≥ 71 g); Larsson 2013 (90 g)	0.93 (0.77, 1.12)	Knekt 2000, Hansen 2017 (M ≥ 54 g, W ≥ 1 g); Larsson 2013 (180 g)	0.92 (0.76, 1.12)				
	(139, 507)		*I* ^2^ = 0.0%		*I* ^2^ = 0.0%				
All-cause mortality	3	Knekt 1996 (M ≥ 54 g, W ≥ 71 g); Hodgson 2016 (>100 g); Roswell 2015 (≥ 35.2 g)	**0.85 (0.77, 0.92)**	Knekt 1996 (M ≥ 54 g, W ≥ 1 g); Hodgson 2016 (5–100 g); Roswell 2015 (≥ 35.2 g)	**0.85 (0.79, 0.92)**				
	(51, 550)		*I* ^2^ = 0.0%		*I* ^2^ = 0.0%				

1 Meta-analyses were conducted using the random-effects model. *I*^2^ is an indicator of between-comparison heterogeneity. *I*^2 ^>50% was deemed as having significant heterogeneity. T2DM, type 2 diabetes mellitus..

##### Acute coronary syndrome

The Danish Diet, Cancer and Health Study cohort (20) followed 55,338 men and women between the ages of 50 and 64 for a median of 7.7 y and reported a significantly decreased risk of acute coronary syndrome (ACS) [incidence rate ratio (IRR): 0.78, 95% CI: 0.64, 0.95] in men with high apple intake. However, no association was observed in women (IRR: 0.93, 95% CI: 0.67, 1.29). Additional analyses found that for every 25 g/d increase in apple intake, men had a decreased risk of ACS (IRR: 0.97, 95% CI: 0.94, 0.99), but women did not (IRR: 0.97, 95% CI: 0.93, 1.01).

##### Cerebrovascular disease

Four studies (17, 21, 40, 41) reported an association between apple intake and cerebrovascular disease, including total and acute stroke. All studies included both men and women, and only 1 study (17) stratified results by men and women (reporting 2 data points).

**FIGURE 4:**
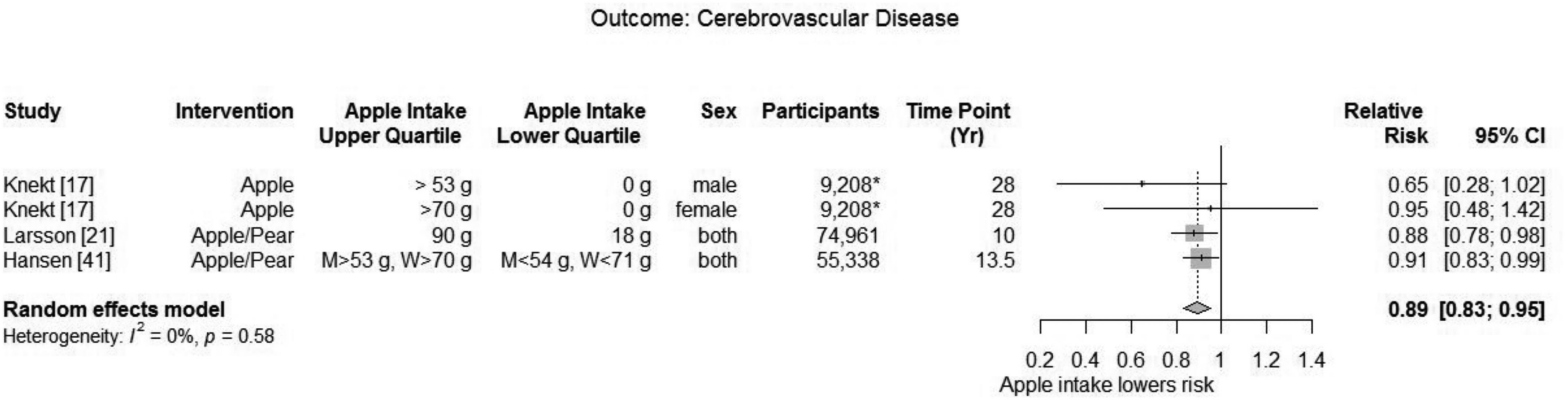
Meta-analysis of observation studies reporting the effect of apple consumption dose on cerebrovascular disease.

Meta-analysis of 3 studies with a total of 139,507 participants found a significantly decreased risk of cerebrovascular disease (summary adjusted RR: 0.89; 95% CI: 0.83, 0.95) without heterogeneity (*I*^2^ = 0.0%; *P* = 0.58) (**Figure 4**). A fourth study (40), conducted in The Netherlands, followed 20,069 participants for 10.3 y and found that each 25 g/d increase in intake of apple was inversely associated with stroke (HR: 0.93; 95% CI: 0.86−1.00).

Meta-analysis of 2 studies (with 3 data points) (17, 21) with a total of 84,169 participants found no difference in risk for cerebral thrombosis or embolism between highest compared with lowest quantiles of apple intake (summary adjusted RR: 0.76; 95% CI: 0.55, 1.06; *I*^2^ = 52.3%; *P* = 0.123).

Meta-analysis of 3 studies [with a total of 139,507 participants (17, 21, 41)] found no difference in risk of intracerebral hemorrhage (summary adjusted RR: 0.92; 95% CI: 0.76, 1.12; *I*^2^ = 0.0%) in subjects at the highest compared with subjects at the lowest quantile of apple intake.

##### Cardiovascular death

Meta-analysis of 4 studies (14, 18, 46, 47) with a total of 36,753 participants found a significant decrease in cardiovascular death (summary adjusted RR: 0.86; 95% CI: 0.78, 0.95) without any heterogeneity (*I*^2^ = 0.0%; *P* = 0.387).

##### All-cause mortality

**FIGURE 5:**
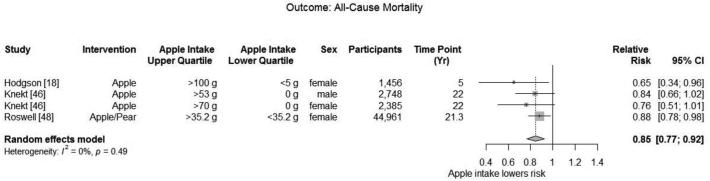
Meta-analysis of observational studies reporting the effect of apple and pear consumption dose on all-cause mortality.

Meta-analysis of 3 studies with a total of 55,625 participants (18, 46, 48) found a significant decrease in all-cause mortality (summary adjusted RR: 0.85; 95% CI: 0.77, 0.92) with low heterogeneity (*I*^2^ = 0.0%; *P* = 0.49) when comparing subjects with the highest intake with subjects with the lowest intake of apple (**Figure 5**). Death was ascertained using data from the Central Statistical Office of Finland (46), the Swedish Bureau of Statistics and Cause of Death Registry (48), or the Western Australian Mortality Database (18).

##### Type 2 diabetes

**FIGURE 6:**
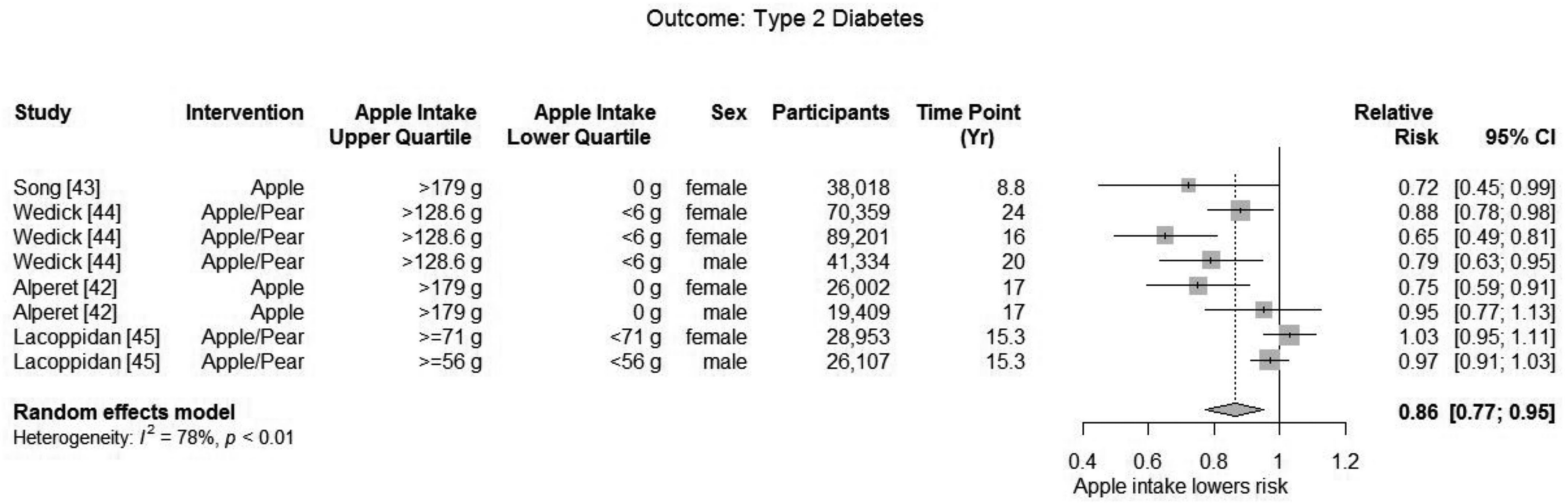
Meta-analysis of observational studies reporting the effect of apple and pear consumption dose on on type 2 diabetes.

Meta-analysis of 6 cohorts in 4 publications (42–45) included 339,383 participants and found a significant decrease in risk of diabetes (summary adjusted RR: 0.86; 95% CI: 0.77, 0.95) with a high heterogeneity (*I*^2^ = 78.0%; *P* = 0.001) when comparing the highest with the lowest quantile of apple or pear intake (**Figure 6**). In sensitivity analysis, 4 cohorts that included only women or that reported results stratified by sex (42–44) also found a significant decrease in risk of diabetes (summary adjusted RR: 0.81; 95% CI: 0.68, 0.96) with a high heterogeneity (*I*^2^ = 89.7%; *P* = 0.001).

Additional sensitivity analyses comparing midquantiles (42–44) to the lowest quantile of apple intake found a significant decrease in the risk of diabetes (summary adjusted RR: 0.88; 95% CI: 0.80, 0.96; *I*^2^ = 84.2%; *P* = 0.001) overall and for a female-only subgroup (summary adjusted RR: 0.86; 95% CI: 0.74, 0.98; *I*^2^ = 87.9%; *P* = 0.001). Meta-analysis comparing medium to low levels of apple intake also found a significant decrease in risk of diabetes (summary adjusted RR: 0.92; 95% CI: 0.86, 0.99; *I*^2^ = 74.9%; *P* = 0.001), but not a significant decrease among the female-only subgroup (summary adjusted RR: 0.92; 95% CI: 0.82, 1.02; *I*^2^ = 81.3%; *P* = 0.001).

##### Body weight

One study (50), comprising 3 prospective cohorts (NHS, NHS II, and HPFS) with a total of 133,468 participants found an association between increased intake of apple and a decrease in body weight by an average of−1.24 lbs (95% CI: −1.62, −0.86) −0.56 kg (95% CI: −0.73, −0.39) after 4 y. In each of the cohorts, following apple intake, body weight decreased an average of−1.43 lbs (95% CI: −1.62, −1.23) −0.65 kg (95% CI: −0.73, −0.56) in NHS, −0.85 lbs (95% CI: −1.06, 0.64) −0.39 kg (−0.48, 0.29) in NHS II, and−1.45 lbs (95% CI: −1.65, −1.25) −0.65 kg (−0.75, −0.57) in HPFS participants.

##### Hypertension

In 1 study, the HRs for incident hypertension comparing the highest and lowest quartiles of apple intake for the NHS, NHS II, and HPFS cohorts were 0.90 (95% CI: 0.85, –0.96, *P* = 0.003), 0.93 (95% CI: 0.87–0.99, *P* = 0.07) and 0.93 (95% CI: 0.86, −1.00, *P* = 0.03), respectively (49). The pooled HR for incident hypertension, comparing the highest quartile with the lowest quartile, was 0.91 (95% CI: 0.88, –0.95, *P* < 0.001).

## Discussion

This systematic review has demonstrated significant reduction in the risk of adverse cardiovascular outcomes associated with apple intake in cohort studies and for the outcome of BMI in RCTs. To the best of our knowledge, this is the first meta-analysis of the impact of apple intake on a comprehensive list of CVD/CMD risk factors and outcomes in cohort studies and in RCTs. Our review found that higher intake of apple, pear, or combined apple and pear was associated with a significant decrease in BMI in RCTs, and a significant decrease in the risk of cerebrovascular disease, CVD/CMD mortality, diabetes, and all-cause mortality in observational studies. There was no difference between apple/pear and control groups for the outcomes of body weight, HDL cholesterol, LDL cholesterol, total cholesterol, and TG in meta-analysis of RCTs, or the risk of cerebral infarction and intracerebral hemorrhage in meta-analysis of cohort studies. While the meta-analysis included between 3 and 5 studies, the number of included participants varied considerably across RCTs and cohort studies. In RCTs, the number of included participants ranged between 175 and 393, and in cohort studies the number of participants ranged between 55,625 and 284,323. Further, individual studies did not find a significant change in waist-to-hip ratio (*n* = 1), apolipoprotein b (*n* = 1), total cholesterol: HDL cholesterol (*n* = 2), glucose: insulin (*n* = 1), insulin (*n* = 3), and VLDL cholesterol (*n* = 1). Studies had conflicting results for LDL cholesterol:HDL cholesterol (*n* = 2), glucose (*n* = 2), SBP (*n* = 2), DBP (*n* = 2), and ACS (*n* = 1).

The protective association observed in this study between apple intake and diabetes is consistent with observations made in a previous systematic review and meta-analysis (23). Though similar, our estimation of the effect of apple intake on diabetes was stronger than that of the previous review (RR of 0.77 compared to RR of 0.82). Unique to this study, we also conducted a sensitivity analysis on cohort studies that only included women and separate meta-analyses of RCTs (23). Our findings are consistent with those of another review that observed protective associations between increased fruit intake and heart disease outcomes (51). However, our review focused solely on apple, pear, or combined apple and pear exposure, while other investigators reviewed studies with a variety of fruit exposures. To the best of our knowledge, our findings of the effect of apple intake on other CVD/CMD risk factors were not addressed in previous systematic reviews and meta-analyses.

The significant association that we demonstrate in this review between apple and pear intake and cardiovascular outcomes in cohort studies is consistent with studies demonstrating the health benefits of apples, possibly due to the presence of phytochemicals like flavonoids, anthocyanins and other antioxidants (13). Apples and pears are a rich source of flavonoids, which have documented cardiovascular benefits (12). The significant association observed between BMI reduction and apple intake in the RCTs is consistent with the results of the meta-analyses of cohort studies in that reduction in BMI correlates strongly with improved cardiovascular outcomes (52). However, we did not observe significant associations among other cardiovascular risk factors with apple intake, including serum lipid concentrations.

In the results reported above, we combined apple and pear intake, because of similarities in nutrient content between apples and pears (53). We examined the validity of this approach in both the RCTs and the cohort studies. In the RCTs, only 1 study that was included in meta-analysis reported results for apple and pear as one of the arms (34). Repeating the meta-analyses for the apple-only subgroup showed a similar trend for the remaining 6 studies that were included in this meta-analysis (**Supplemental Table 6**). Nine out of 14 cohort studies grouped apple and pear intake together into 1 exposure category. When we conducted a subgroup analysis of the 5 studies that studied only apple intake, meta-analyses could be performed only for T2DM incidence and all-cause mortality. For the other outcomes, there were not enough studies available for apple-only analyses. For the 2 outcomes mentioned, the results had similar trends (**Supplemental Table 5**) compared to the results obtained when including studies where apple and pear intake was combined.

In the meta-analyses reported for cohort studies above, we combined studies regardless of whether the studies controlled for diet or not. However, when we performed a subgroup analysis using only those studies that controlled for diet, the results showed trend similar to that seen when all studies were included (**Supplemental Table 7**).

The limitations of this review are largely reflective of the quality of primary studies as well as the consistency of data collection and analysis methods in individual studies. First, many of the included observational studies used data collection and outcome assessment methods that are prone to biases, including self-report for dietary and outcome assessment as well as record linkage for outcome assessment (54). Second, the populations examined in some of the included cohort studies were restricted to nurses or other health professionals who may not be representative of the average individual, potentially limiting the generalizability of the results. There is also a potential for a substitution effect in that subjects who consumed more apples may also have a diet that may substantially vary from other subjects who do not consume apples or other plant-based foods. We observed that in the cohort studies, we have done an analysis controlling for diet where we found the trends to be similar even after controlling for diet. In the randomized clinical trials, the assumption was made that the diets in the various arms were equivalent. Nevertheless, this substitution effect may be a limitation of this meta-analysis. While the majority of cohort studies controlled for diet and lifestyle variables in their analyses, the effect of dietary patterns or overall fruit intake may have confounded the associations from the few studies which did not control for these variables. Lastly, some RCTs failed to describe randomization techniques, varied in the types of controls used to compare the effects of apple intake, and failed to provide sufficient information regarding participants’ compliance with the intervention.

## Conclusion

Except for a significant decrease in BMI, meta-analyses of apple or pear intake in RCTs did not show a statistically significant change in risk factors for CVD/CMD. Observational studies demonstrated an association between apple, pear, or combined apple and pear intake and a decreased risk of acute coronary disease, cerebrovascular disease, CVD mortality, diabetes, and all-cause mortality suggesting that apple intake may be of benefit in CVD/CMD prevention.

## Supplementary Material

nzz109_Supplement_File

## Notes

An abstract on this research was presented at the 2018 American Society for Nutrition conference.

Supported by a grant from the US Apple Association.

The funder did not have a role in the study selection, quality assessment, data synthesis, or manuscript preparation.

BAG, EEA, EE, NN, EJJ, and GR, no conflicts of interest.

Supplemental Tables 1–7 and Supplemental Figure 1 are available from the “Supplementary data” link in the online posting of the article and from the same link in the online table of contents at https://academic.oup.com/cdn/.

Abbreviations used: ACS, acute coronary syndrome; CMD, cardiometabolic disease; CVD, cardiovascular disease; DBP, diastolic blood pressure; IRR, incidence rate ratio; QALY, quality adjusted life year; RCT, randomized controlled trial; SBP, systolic blood pressure; T2DM, type 2 diabetes mellitus.
