# Supplementary material for: Effects of Intake of Apples, Pears, or Their Products on Cardiometabolic Risk Factors and Clinical Outcomes: A Systematic Review and Meta-Analysis
Source: Curr Dev Nutr. 2019 Oct 3;3(10):nzz109. doi: 10.1093/cdn/nzz109 (PMC6813372; doi:10.1093/cdn/nzz109)
Supplement: nzz109_Supplement_File [file nzz109_supplement_file.docx]

“Effect of intake of apples, pears, or their products on cardiometabolic risk factors and clinical outcomes: a systematic review and meta-analysis”

Gayer

Online Supplementary Material

**Supplemental Table 1.** Search conducted in MEDLINE and Cochrane Central databases

|  | **Searches** |
| --- | --- |
| 1 | exp malus/ or malus.mp. or apple.tw. or apples.tw. or (apple and juice).tw. |
| 2 | (cardiovascular or cardiac).tw. or Cardiovascular Diseases/ep or Cardiovascular Diseases/et or Cardiovascular Diseases/pc or Cardiovascular Diseases/mo |
| 3 | heart.tw. or Heart Diseases/ep or Heart Diseases/et or Heart Diseases/pc or Heart Diseases/mo |
| 4 | (myocardial and (infarction or ischemia)).tw. or myocardial ischemia/et or myocardial ischemia/ep or myocardial ischemia/pc or myocardial ischemia/mo |
| 5 | Stroke$.tw. or Stroke/et or Stroke/ep or Stroke/pc or Stroke/mo |
| 6 | (coronary and (artery or arteriosclerosis or atherosclerosis)).tw. |
| 7 | exp coronary artery disease/ or exp coronary heart disease/ |
| 8 | (heart attack$ or sudden death$).tw. |
| 9 | cerebral.tw. or Cerebral Infarction/et or Cerebral Infarction/ep or Cerebral Infarction/pc or Cerebral Infarction/mo |
| 10 | exp cerebrovascular disorder/ or cerebrovascular disorders/et or cerebrovascular disorders/ep or cerebrovascular disorders/pc or cerebrovascular disorders/mo or cerebrovascular.tw. |
| 11 | Heart failure/ep or Heart failure/et or Heart failure/pc or Heart failure/mo |
| 12 | Metabolic Syndrome X/ep or Metabolic Syndrome X/et or Metabolic Syndrome X/pc |
| 13 | ((serum adj1 cholesterol) or (dyslipid$ or hypolipid$ or hyperlipid$) or (LDL or VLDL or triglyceride$ or HDL)).tw. |
| 14 | (Blood pressure or hypertension).af. or exp hypertension/ |
| 15 | exp diabetes mellitus/ or diabetes.af. |
| 16 | exp blood glucose/ or (blood and glucose).af. or blood glucose/ |
| 17 | exp insulin/ or insulin.af. or HOMA.af. |
| 18 | exp body weight/ or (body and weight).af. |
| 19 | exp body mass index/ or BMI.tw. or (body and mass and index).af. |
| 20 | exp waist-hip ratio/ or (waist-hip and ratio).af. |
| 21 | exp Atrial Fibrillation/ or exp Peripheral Vascular Diseases/ or exp vascular disease/ or angina.af. |
| 22 | exp mortality/ or mortality.af. |
| 23 | or/2-22 |
| 24 | 1 and 23 |
| 25 | Animal/ not Human/ |
| 26 | 24 not 25 |
| 27 | review$.pt. |
| 28 | 26 not 27 |
| 29 | remove duplicates from 28 |

**Supplemental Table 2.** Risk of Bias of intervention studies

**
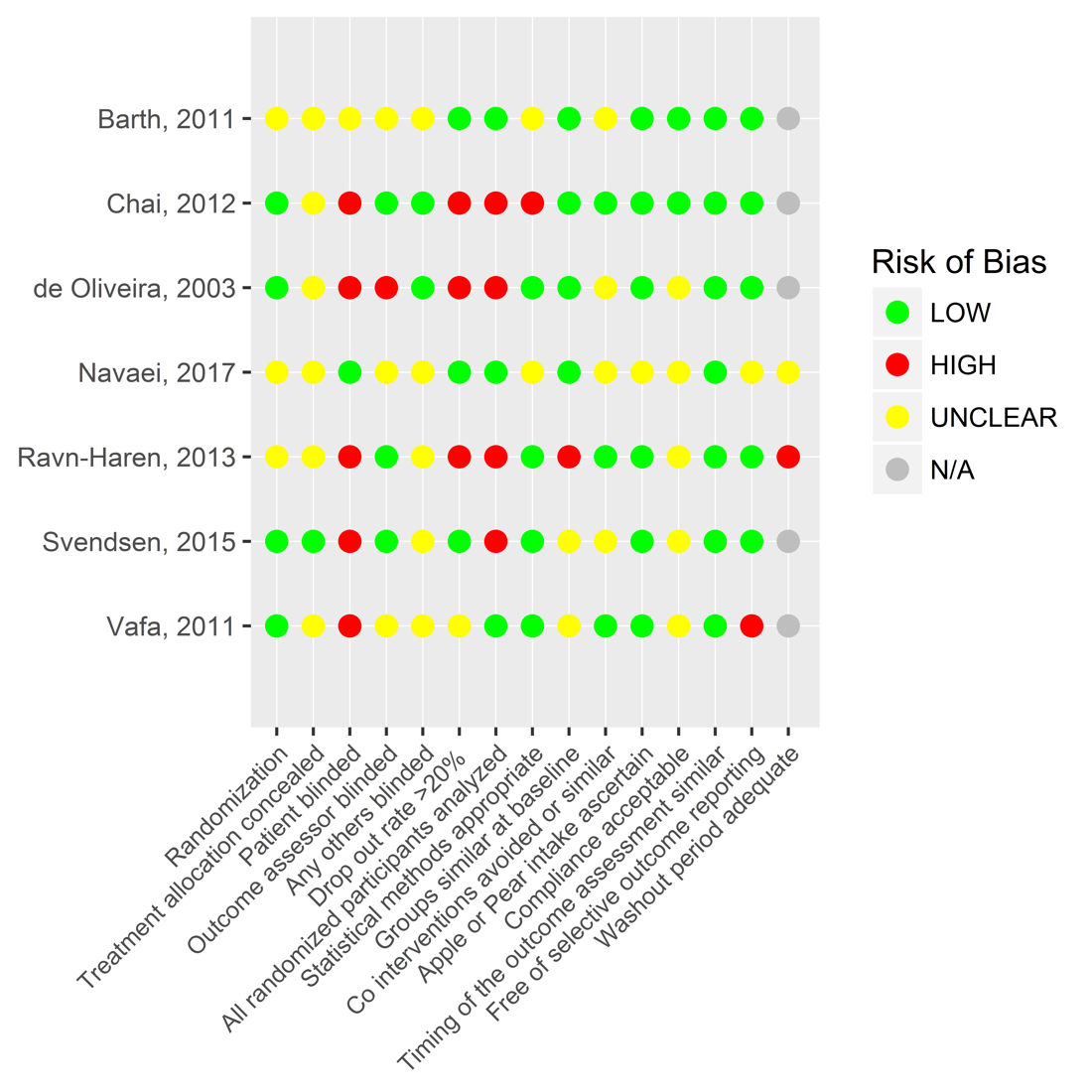
**

**Supplemental Table 3.**Meta-analysis of **% Net change** of Trials Reporting the Effect of Apple versus low dose on Serum Lipids and Body Weight

| Outcome | N Studies  (N Subjects) | Analysis 1  Net change (95%CI) |
| --- | --- | --- |
| Total Cholesterol (mg/dL) | 4* | -0.61 (-4.25, 3.04) |
|  | (289) | I^2^ = 0.0% |
| LDL-C (mg/dL) | 4 | -0.21 (-4.95, 4.54) |
|  | (289) | I^2^ = 0.0% |
| HDL-C (mg/dL) | 4 | -0.18 (-4.61, 4.26) |
|  | (289) | I^2^ = 0.0% |
| Triglycerides (mg/dL) | 5 | 1.99 (-6.65, 10.63) |
|  | (324) | I^2^ = 0.0% |
| Body Weight (kg) | 4* | 0.10 (-4.11, 3.92) |
|  | (347) | I^2^ = 0.0% |
| BMI (kg/m^2^) | 3 | 0.01 (-4.73, 4.76) |
|  | (229) | I^2^ = 0.0% |

Meta-analyses were conducted using the random-effects model.

Analysis 1: For one 3-arm parallel trial Chai 2012, the main analyses included 3 month intake time-point because it was the closest to the final time points in the other studies.

I^2^ is an indicator of between-comparison heterogeneity. I^2^ >50% was deemed as having significant heterogeneity.

BMI = Body Mass Index; HDL-C = High-density lipoprotein cholesterol; LDL-C = Low-density lipoprotein cholesterol; N = number; N/A = not applicable.

*Omitted a study due to lack of enough data to calculate % net change.

**Supplemental Table 4.** Risk of Bias of Cohort studies*


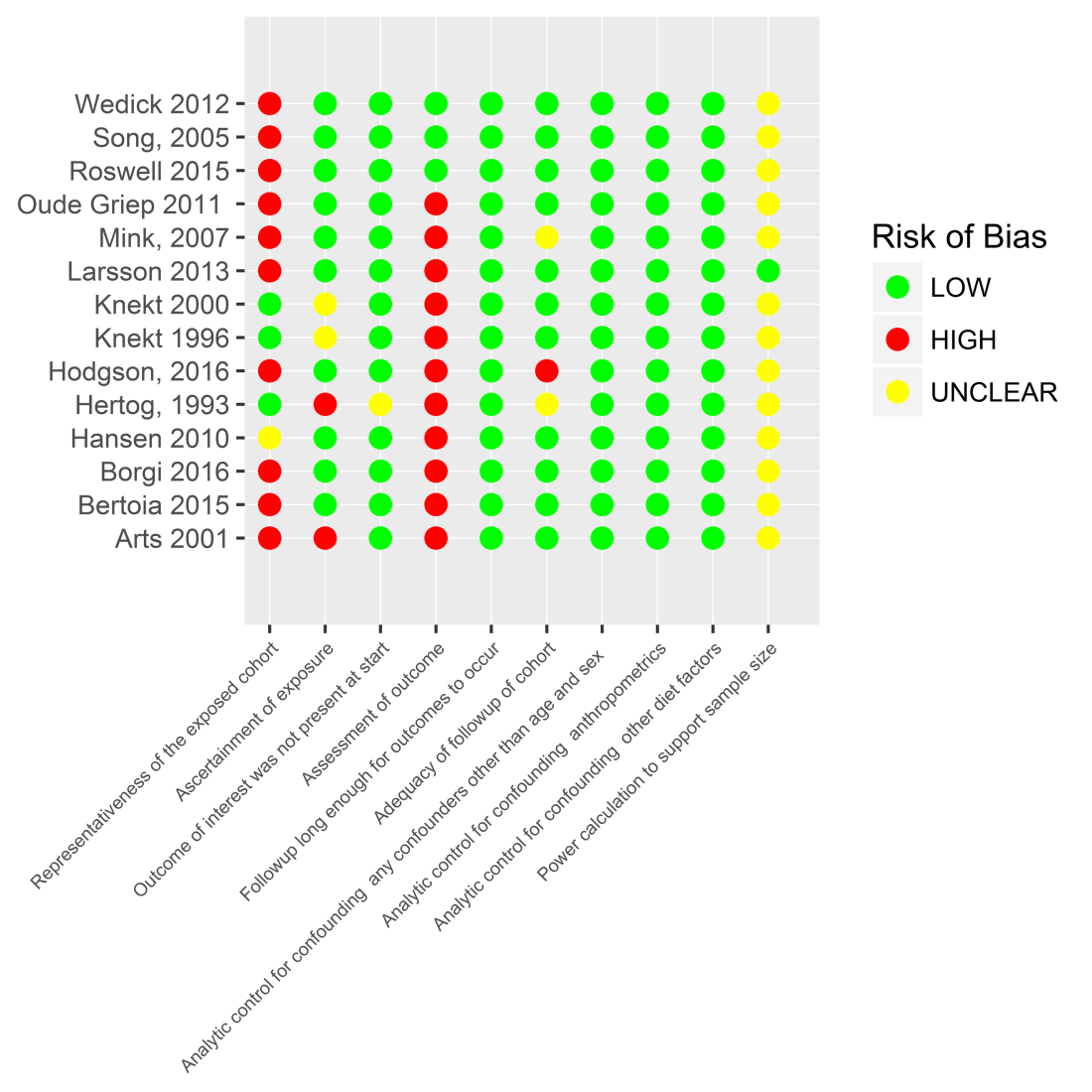


*In Hodgson 2016, Assessment of Outcome had a LOW risk of bias for all-cause mortality and a HIGH risk of bias for CVD mortality

** The Danish Diet, Cancer and Health cohort reported in Hansen 2010 was also reported in Hansen 2017 and Lacoppidan 2015

**Supplemental Table 5.** Meta-analysis of Observation Studies comparing the meta-analyses of apple and pear vs. apple only studies.

| **Outcome** | **Apple + Pear Studies** | **Apple + Pear**  **meta-analysis** | **Apple-only studies** | **Apple-only subgroup meta-analysis** |
| --- | --- | --- | --- | --- |
| **Cerebrovascular disease/Total stroke** | Knekt 2000;  Hansen 2017 (M>=54g, W>=71g);  Larsson (90g) | 0.89 (0.83, 0.95);  I^2^ = 5.5% | Knekt 2000 (Men, Women) | Not enough studies. |
| **Cardiovascular death** | Hertog 1993 (>110g); Hodgson 2016 (>100g); Mink (>20 g) | 0.86 (0.78, 0.95);  I^2^ = 0.0% | Hertog 1993;  Hodgson 2016 | Not enough studies. |
| **Thrombosis or embolia or CI** | Knekt 2000 (M>=54g, W>=71g);  Larsson 2013 (90 g) | 0.76 (0.55, 1.05);  I^2^ = 50.4% | Knekt 2000 (Men, Women) | Not enough studies. |
| **Type 2 Diabetes Incidence** | Alperet 2017; Lacoppidan 2015;  Song 2005 (>=180);  Wedick (>128.6) | 0.86 (0.77, 0.95);  I^2^ = 78.8% | Song 2005;  Alperet 2017 (Men, Women) | 0.81 (0.68, 0.96);  I^2^ = 59.8% |
| **Type 2 Diabetes Incidence (Females-only)** | Alperet 2017; Lacoppidan 2015 (>71);  Song 2005 (>=180);  Wedick (>128.6) | 0.81 (0.68, 0.96);  I^2^ = 789.7% | Song 2005;  Alperet 2017 (Women) | Not enough studies. |
| **Intracerebral haemorrhage** | Knekt 2000;  Hansen 2017 (M>=54g, W>=71g);  Larsson 2013 (90 g) | 0.93 (0.77, 1.12);  I^2^ = 0.0% | Knekt 2000 (Men, Women) | Not enough studies. |
| **All-cause mortality** | Knekt 1996 (M>=54g, W>=71g);  Hodgson 2016 (>100 g); Roswell 2015 (>=35.2g) | 0.83 (0.74, 0.92);  I^2^ = 27.8% | Hodgson 2016;  Knekt 2000 (Men, Women) | 0.78 (0.68, 0.89);  I^2^ = 4.7% |

**Supplemental Table 6: Results for RCT subgroup analysis without apple/pear study**

|  | **N Studies (N Subjects)** | **Analysis1: Net change (95%CI)** | **Analysis 2: Net change (95%CI)** | **Analysis 3: Net change (95%CI)** |
| --- | --- | --- | --- | --- |
| **Total Cholesterol (mg/dL)** | 4 (289) | -6.09 (-14.04, 1.85) | -7.95 (-18.75, 2.85) | -6.83 (-16.09, 2.42) |
|  |  | I^2^ = 34.2% | *I^2^ = 63.3%* | *I^2^ = 49.1%* |
| **LDL-C (mg/dL)** | 4 (289) | No apple & pear | No apple & pear | No apple & pear |
|  |  | studies in analysis | studies in analysis | studies in analysis |
| **HDL-C (mg/dL)** | 4 (289) | No apple & pear | No apple & pear | No apple & pear |
|  |  | studies in analysis | studies in analysis | studies in analysis |
| **Triglycerides (mg/dL)** | 4 (289) | 4.94 (-13.03, 22.91) | 5.86 (-11.53, 23.25) | 3.77 (-18.99, 26.54) |
|  |  | *I^2^ = 58.2%* | *I^2^ = 52.4%* | *I^2^ = 73.8%* |
| **Body Weight (kg)** | 4 (359) | 0.14 (-0.45, 0.74) | 0.14 (-0.45, 0.73) | 0.14 (-0.45, 0.74) |
|  |  | I^2^ = 0.0% | I^2^ = 0.0% | I^2^ = 0.0% |
| **BMI (kg/m^2^)** | 2(194) | Not enough studies. | Not enough studies. | Not enough studies. |

Meta-analyses were conducted using the random-effects model.

Analysis 1: For one 3-arm parallel trial Chai 2012, the main analyses included 3 month intake time-point because it was the closest to the final time points in the other studies.

Analysis 2: For one 3-arm parallel trial Chai 2012, sensitivity analysis was conducted using the 6 month time point

Analysis 3: For one 3-arm parallel trial Chai 2012, sensitivity analysis was conducted using the 12 month time point

I^2^ is an indicator of between-comparison heterogeneity. I^2^ >50% was deemed as having significant heterogeneity.

BMI = Body Mass Index; HDL-C = High-density lipoprotein cholesterol; LDL-C = Low-density lipoprotein cholesterol; N = number; N/A = not applicable.

**Supplemental Table 7:Results for Cohort subgroup analysis excluding studies without diet-adjusted analyses**

|  | **N Studies**  **(N Subjects)** | **Analysis 1 High dose** | **Original Analysis 1**  **Net change (95%CI)** | **Publication removed** | **Subgroup analysis results** |
| --- | --- | --- | --- | --- | --- |
| **Cerebrovascular disease/ Total stroke** | 3 | Knekt 2000, Hansen 2017; Larsson 2013 | 0.89 (0.83, 0.95) | Larsson 2013 | 0.86 (0.71, 1.04) |
|  | (139,507) |  | I^2^ = 5.5% |  | I^2^ = 35.5% |
| **Thrombosis or embolia or CI** | 2 | Knekt 2000; Larsson 2013 | 0.76 (0.55, 1.05) | Larsson 2013 | Not enough |
|  | (84,169) |  | I^2^ = 50.4% |  | Studies left |
| **Intracerebral haemorrhage** | 3 | Knekt 2000; Hansen 2017; Larsson 2013 | 0.93 (0.77, 1.12) | Larsson 2013 | 0.93 (0.73, 1.19) |
|  | (139,507) |  | I^2^ = 0.0% |  | I^2^ = 0.0% |
| **All-cause mortality** | 3 | Knekt 1996; Hodgson 2016 Roswell 2015 | 0.83 (0.74, 0.92) | Knekt 1996 | Not enough |
|  | (51,550) |  | I^2^ = 27.8% |  | studies left |

**Supplement Figure 1**: Analytical framework describing potential association between apple/pear intake and CVD risk factors and outcomes


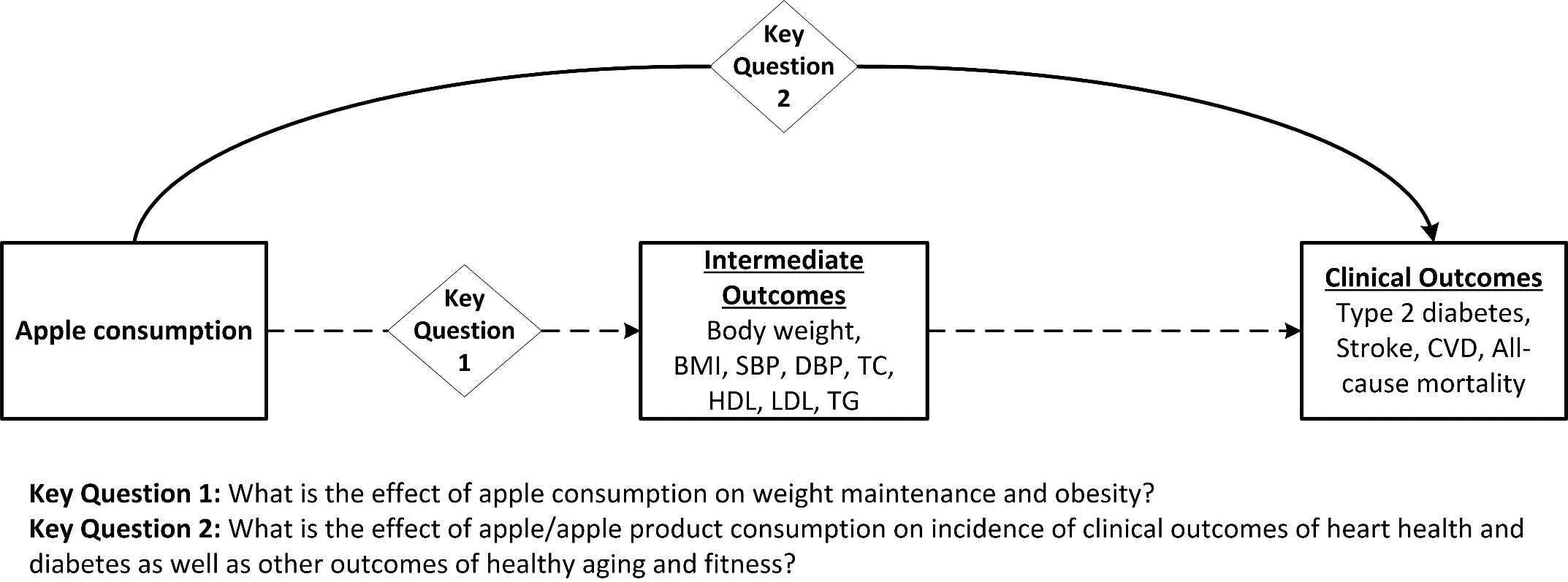


**Intake of apple, pear and their products**
